# What motivates gambling behavior? Insight into dopamine's role

**DOI:** 10.3389/fnbeh.2013.00182

**Published:** 2013-12-02

**Authors:** Patrick Anselme, Mike J. F. Robinson

**Affiliations:** ^1^Département de Psychologie, Université de LiègeLiège, Belgium; ^2^Department of Psychology, University of MichiganMichigan, MI, USA; ^3^Department of Psychology, Wesleyan UniversityConnecticut, CT, USA

**Keywords:** dopamine, motivation, gambling, loss, reward uncertainty

It is commonly believed that monetary gain is the cause of gambling behavior in humans. Mesolimbic dopamine (DA), the chief neuromediator of incentive motivation, is indeed released to a larger extent in pathological gamblers (PG) than in healthy controls (HC) during gambling episodes (Linnet et al., 2011; Joutsa et al., 2012), as in other forms of compulsive and addictive behavior. However, recent findings indicate that the interaction between DA and reward is not so straightforward (Blum et al., 2012; Linnet et al., 2012). In PG and HC, DA release seems to reflect the unpredictability of reward delivery rather than reward *per se*. This suggests that the motivation to gamble is strongly (though not entirely) determined by the inability to predict reward occurrence. Here we discuss several views of the role of DA in gambling, and attempt to provide an evolutionary framework to explain its role in uncertainty.

## Traditional view: money drives gambling

Common sense suggests that if gambling at casinos is attractive for many people, it is because it offers an opportunity to win money (Dow Schüll, 2012). Of course, a “big win” is rare, but the random component behind most games and the publicizing of big winners lets people believe that the chance of winning a lot is not so unlikely. In this traditional view, money is a gambler's primary motivation, and randomness in games allows the gambler to hope that the gains will overcome the losses.

This view is compatible with the evidence that DA released in the nucleus accumbens, a mesolimbic region in the brain, magnifies the attractiveness of rewards and conditioned cues (Berridge, 2007). Mesolimbic DA transforms neutral cues into conditioned cues when they come to reliably predict reward delivery (Melis and Argiolas, 1995; Peciña et al., 2003; Flagel et al., 2011). Money is certainly a strong conditioned cue, which has been associated with abundance and power in all human civilizations. As with other reward sources, money is known to enhance mesolimbic DA levels in the human striatum during gambling episodes, suggesting that money is what motivates gamblers (Koepp et al., 1998; Zald et al., 2004; Zink et al., 2004; Pessiglione et al., 2007). For example, Joutsa et al. (2012) showed that DA is released in the ventral striatum during instances of high- but not low-reward, in both PG and HC, and that the severity of symptoms in PG is associated with larger DA responses.

## The attractiveness of losses

Although the traditional view is in agreement with neuroscientific data, it fails to explain why people often describe gambling as a pleasant activity rather than as an opportunity to gain money. During gambling episodes, PG report euphoric feelings comparable to those experienced by drug users (van Holst et al., 2010), and the more PG lose money, the more they tend to persevere in this activity—a phenomenon referred to as loss-chasing (Campbell-Meiklejohn et al., 2008). Such results are hardly compatible with the traditional view. Animal and human studies indicate that the role of DA in reward is, at least in gambling, more complex than initially believed (Linnet, 2013).

Determining the exact timing of subjective feelings or how losses spur on a gambler's desire to play during gambling episodes is difficult because different emotions and cognitions constantly overlap. Nevertheless, Linnet et al., (2010) were able to measure mesolimbic DA release in PG and HC winning or losing money. Unexpectedly, they found no difference in dopaminergic responses between PG and HC who won money. Dopamine release in the ventral striatum, however, was more pronounced for the losses in PG relative to HC. Given the motivational impact of mesolimbic DA, Linnet and colleagues argue that this effect could explain loss-chasing in PG. In addition, they point out that “PG are not hyperdopaminergic *per se*, but have increased DA susceptibility toward certain types of decisions and behavior” (p. 331). This finding that DA release is higher in PG losing money than in PG winning money is consistent with the evidence that “near misses” enhance the motivation to gamble and recruit the brain reward circuit more than “big wins” (Kassinove and Schare, 2001; Clark et al., 2009; Chase and Clark, 2010). Possibly related to this phenomenon is the evidence that, compared with gains, the amount of monetary losses has limited effect on the extent to which probabilistic (and delayed) losses are discounted in humans (Estle et al., 2006). This suggests that a lower probability (and a longer delay) reduces a gambler's motivation less when losses rather than gains are involved. In contrast, the big win hypothesis suggests that pathological gambling develops in individuals that initially experienced large monetary gains, but the attempts to demonstrate this effect on persistence of gambling have failed (Kassinove and Schare, 2001; Weatherly et al., 2004). Current evidence therefore suggests that losses contribute to motivate gambling more than gains.

## The attractiveness of reward uncertainty

One of the main underlying factors to the phenomenon of loss-chasing may relate to the importance of reward uncertainty. Studies have shown that reward uncertainty rather than reward *per se*, will magnify mesolimbic DA, both in monkeys (Fiorillo et al., 2003; de Lafuente and Romo, 2011) and healthy human participants (Preuschoff et al., 2006). In PG, accumbens DA is maximal during a gambling task when the probability of winning and losing money is identical—a 50% chance for a two-outcome event representing maximal uncertainty (Linnet et al., 2012). Although non-dopaminergic neurons might also be involved in the coding of reward uncertainty (Monosov and Hikosaka, 2013), these results based on electrophysiological and neuroimaging techniques indicate that DA is crucial for the coding of reward uncertainty. This suggestion is corroborated by a large number of behavioral studies, showing that mammals and birds respond more vigorously to conditioned cues predicting uncertain rewards (Collins et al., 1983; Anselme et al., 2013; Robinson et al., under review) and tend to prefer an uncertain food option over a certain food option in dual-choice tasks (Kacelnik and Bateson, 1996; Adriani and Laviola, 2006), sometimes despite a lower reward rate (Forkman, 1991; Gipson et al., 2009). According to Greg Costikyan, an award-winning game designer, games cannot hold our interest in the absence of uncertainty—which can take many forms, occurring in the outcome, the game's path, analytical complexity, perception, and so on (Costikyan, 2013). Discussing the game of *Tic-Tac-Toe*, Costikyan (p. 10) notes that this game is dull for anyone beyond a certain age because its solution is trivial. The reason why children play this game with enjoyment is that they do not understand that the game has an optimal strategy; for children, the game of *Tic-Tac-Toe* produces an uncertain outcome. A predictable game is dull, just like a detective novel for which the identity of the murderer is known in advance. Based on this assumption, Zack and Poulos (2009) note that several payoff schedules (slot machines, roulette, and dice game of craps) have a probability of winning close to 50%, so that they are expected to elicit maximal DA release and, therefore, reinforce the act of gambling.

The evidence that uncertainty itself appears to be a source of motivation is visible in the growing trend of pathological gambling that involves extended play at video poker or slot machines (Dow Schüll, 2012). Individuals are playing to play rather than to win, and monetary wins are conceived as the opportunity to extend the duration of play, rather than the game's main objective. In addition, game programmers have uncovered a profitable trend toward larger and larger number of bets per round of a given game (in Australia, >100 bets on a given roll), with smaller and smaller amounts (going as low as one cent), giving rise to a “losses disguised as wins” effect, where players win less than they wagered (Dixon et al., 2010). It is almost as if players were drawn to placing bets or trying to uncover the algorithm that determines the wins and losses (this is often reported in players, see Dow Schüll, 2012). Recently, we have shown in adult rats that an initial exposure (8 days) to conditioned cues predicting highly uncertain rewards sensitizes responding to those cues in the long term (for at least 20 days) despite a gradual reduction in the level of uncertainty (Robinson et al., under review). No behavioral sensitization was apparent following a later exposure to high uncertainty (rewards were provided with certainty during the first 8 days). This result is compatible with other findings showing that persistent gambling behavior is more likely to occur in individuals that experience unpredictable environments and gambling situations early in life (Scherrer et al., 2007; Braverman and Shaffer, 2012).

## A possible evolutionary origin of gambling behavior

Since wins are rare and often small during gambling episodes, it is unlikely that they are sufficient to motivate people to persevere in the task. The fact that losses can motivate gambling more than gains is also difficult to understand. So, why do people gamble? Pathological gambling is certainly maladaptive behavior, but the attractiveness of uncertain rewards is so widespread in the animal kingdom that this tendency should have an adaptive origin. Here we suggest a hypothesis—referred to as the compensatory hypothesis—developed by one of the authors, that describes gambling-like behavior in an evolutionary framework (Anselme, 2013).

In nature, animals are subject to a lack of cognitive control in many circumstances; they are often unable to predict what is going to happen. This essentially occurs for two reasons. First, the distribution of natural resources is random, so that a large number of responses must be produced before finding vital resources. Second, the reliability of conditioned cues is often imperfect—e.g., for some species, fruit-trees may act as conditioned cues because of their association with reward (the presence of fruits), but this association is unreliable since fruit-trees have no fruits for most of the year. Given this lack of cognitive control about objects and events, it can be argued that if reward uncertainty were not a source of motivation, most behaviors would extinguish because of the high failure rate (and energy loss) experienced by animals. The compensatory hypothesis suggests that, when a significant object or event's predictability is low, motivational processes are recruited to compensate for the inability to make correct predictions; motivation would act as a mechanism to delay extinction (Anselme, 2013). In other words, allowing an animal to persevere in a task is only possible if its behavior is motivated by the lack of predictability (i.e., uncertainty) rather than by reward itself. The compensatory hypothesis could explain why losses are so important in motivating human gamblers: without the opportunity of receiving no reward, gains become predictable and hence most games become dull (Costikyan, 2013). In addition, this hypothesis provides an interpretation to the evidence that, like physiological deprivations (Nader et al., 1997), psychosocial deprivations such as a lack of maternal care enhance mesolimbic DA release and, correlatively, incentive motivation to seek food (Lomanowska et al., 2011). Psychosocial deprivations also seem to be a cause of gambling-like behavior in both pigeons and humans (van Holst et al., 2010; Pattison et al., 2013). In fact, all forms of deprivation result from the inability to predict how to find/obtain appropriate stimuli—whether food, social relationships, opportunities to work and play, etc. In most cases, this inability is a consequence of environmental poverty. On account of this, poor environments resemble unpredictable environments and the compensatory hypothesis suggests that, in both cases, a higher motivation is recruited to persevere in the laborious task of finding resources.

Assuming that this interpretation is correct, gambling behavior in humans could be phylogenetically inherited from older mammalian species whose members motivated by reward uncertainty had a better chance of survival in complex, dynamic environments. Pathological gambling might be the exaggeration of a natural tendency exploited by casinos and games of chance. Of course, uncertainty-driven motivation is no longer required to survive within most western cultures. However, gambling might be hijacking an evolutionary system designed to resolve uncertainty by spurring pulses of motivation, despite or because of repeated losses. How could pathological gambling be addressed? We think that this psychopathology should certainly be treated on a case-by-case basis, depending on the vulnerability of each PG. For example, favoring enrichment of a PG's daily environment by varying leisure activities and social relations may reduce his desire to seek a surplus of stimulation. At a societal level, one approach allowing to address pathological gambling might be that gamblers at casinos can win more often than they lose but only very small gains (similar to the wagered amounts) in order to render gambling persistence less attractive. More thorough investigations are needed to identify the parameters underpinning the addictive power of games and to promote the development of games which do not exploit our phylogenetic vulnerability.
